# Education intervention for the elderly preparing for end-of-life

**DOI:** 10.1017/S1478951525100758

**Published:** 2025-09-05

**Authors:** Septinda Rima Dewanti

**Affiliations:** Department of Educational Psychology and Guidance, Yogyakarta State University, Yogyakarta, Indonesia

**Dear Editor**

I recently reviewed the article published in your journal titled “Effectiveness of an educational intervention in enhancing end-of-life care understanding and decision-making in African Americans” by Pruitt et al. (2025). The article addresses a critical subject of enhancing preparedness among adults for end-of-life care. This educational intervention is significant not only for adults and the elderly but also for their families and discussing strategies to prepare for the end-of-life stage is highly pertinent in the context of contemporary lifestyles.

Article by Pruitt et al. (2025) advocates for education targeted at the elderly, involving professionals like counsellors and physicians, to increase their awareness and readiness for the end-of-life stage. A recent study employing reflexive thematic analysis discovered that the nature of communication between patients and healthcare professionals can influence their level of trust, sometimes making patients more trusting of their healthcare providers than their family (Sunde et al. 2025). So, it is clear that involving healthcare providers in educating the elderly about end-of-life is beneficial.

In light of the significance of educational interventions as delineated by Pruitt et al. (2025), this correspondence aims to offer supplementary considerations regarding the implementation of educational interventions for geriatric patients. Specifically, it is proposed that such interventions should not solely emphasize cognitive knowledge but also enhance patients’ confidence in decision-making. Furthermore, it is recommended that family members be actively involved in the educational process. These additional considerations contribute to the novelty of this study.

A scoping review of education for adult patients and families with brain injury found that the education programs should specifically focus on not only elderly knowledge, but also confidence in one’s ability to handle problems (i.e. self-efficacy) (Hart et al. 2018). They also found that a greater degree of investment in one’s rehabilitation should be the goal of educating adult patients. Their study suggests that these two outcomes, as gains in these areas, have the potential to “snowball” into additional improvements in functioning and emotional well-being of the patients (Hart et al. 2018).

An additional factor to consider in delivering education to elderly patients is the involvement of family members. A study conducted by Sunde et al. (2025) highlights the necessity for healthcare services to account for the competence and time required to balance the dual responsibilities of providing optimal care for older adults while attending to the needs of family caregivers. Furthermore, evidence indicates that informed and supported families tend to play a more active and effective role as caregivers, which is advantageous for the patient (Park et al. 2018; Smith et al. 2024).

Although engaging caregivers and family members in the care of elderly patients is crucial, it is observed that elderly individuals might refrain from discussing health issues with their families to avert causing concern or stress (Sunde et al. 2025). Consequently, it is imperative to provide education and informational resources to caregivers.
